# Comparison of Four Machine Learning Techniques for Prediction of Intensive Care Unit Length of Stay in Heart Transplantation Patients

**DOI:** 10.3389/fcvm.2022.863642

**Published:** 2022-06-21

**Authors:** Kan Wang, Li Zhao Yan, Wang Zi Li, Chen Jiang, Ni Ni Wang, Qiang Zheng, Nian Guo Dong, Jia Wei Shi

**Affiliations:** ^1^Department of Cardiovascular Surgery, Union Hospital, Tongji Medical College, Huazhong University of Science and Technology, Wuhan, China; ^2^Department of Hand Surgery, Union Hospital, Tongji Medical College, Huazhong University of Science and Technology, Wuhan, China; ^3^Department of Gastroenterology, Union Hospital, Tongji Medical College, Huazhong University of Science and Technology, Wuhan, China; ^4^Department of Nurse, Jianshi County People’s Hospital, Enshi, China

**Keywords:** heart transplantation, ICU-LOS, AUC-ROC, machine learning, XGboost, SHAP (Shapley Additive explanations)

## Abstract

**Background:**

Post-operative heart transplantation patients often require admission to an intensive care unit (ICU). Early prediction of the ICU length of stay (ICU-LOS) of these patients is of great significance and can guide treatment while reducing the mortality rate among patients. However, conventional linear models have tended to perform worse than non-linear models.

**Materials and Methods:**

We collected the clinical data of 365 patients from Wuhan Union Hospital who underwent heart transplantation surgery between April 2017 and August 2020. The patients were randomly divided into training data (*N* = 256) and test data (*N* = 109) groups. 84 clinical features were collected for each patient. Features were validated using the Least Absolute Shrinkage and Selection Operator (LASSO) regression’s fivefold cross-validation method. We obtained Shapley Additive explanations (SHAP) values by executing package “shap” to interpret model predictions. Four machine learning models and logistic regression algorithms were developed. The area under the receiver operating characteristic curve (AUC-ROC) was used to compare the prediction performance of different models. Finally, for the convenience of clinicians, an online web-server was established and can be freely accessed *via* the website https://wuhanunion.shinyapps.io/PredictICUStay/.

**Results:**

In this study, 365 consecutive patients undergoing heart transplantation surgery for moderate (NYHA grade 3) or severe (NYHA grade 4) heart failure were collected in Wuhan Union Hospital from 2017 to 2020. The median age of the recipient patients was 47.2 years, while the median age of the donors was 35.58 years. 330 (90.4%) of the donor patients were men, and the average surgery duration was 260.06 min. Among this cohort, 47 (12.9%) had renal complications, 25 (6.8%) had hepatic complications, 11 (3%) had undergone chest re-exploration and 19 (5.2%) had undergone extracorporeal membrane oxygenation (ECMO). The following six important clinical features were selected using LASSO regression, and according to the result of SHAP, the rank of importance was (1) the use of extracorporeal membrane oxygenation (ECMO); (2) donor age; (3) the use of an intra-aortic balloon pump (IABP); (4) length of surgery; (5) high creatinine (Cr); and (6) the use of continuous renal replacement therapy (CRRT). The eXtreme Gradient Boosting (XGBoost) algorithm presented significantly better predictive performance (AUC-ROC = 0.88) than other models [Accuracy: 0.87; sensitivity: 0.98; specificity: 0.51; positive predictive value (PPV): 0.86; negative predictive value (NPV): 0.93].

**Conclusion:**

Using the XGBoost classifier with heart transplantation patients can provide an accurate prediction of ICU-LOS, which will not only improve the accuracy of clinical decision-making but also contribute to the allocation and management of medical resources; it is also a real-world example of precision medicine in hospitals.

## Introduction

Nowadays, heart transplantation surgery is a life-saving last resort for patients with terminal heart disease (1). The number of heart failure patients is rapidly increasing in developed countries due to population ageing (2). As the largest developing country and the second-largest economy in the world, China is no exception (3). It is estimated that there are over 4.5 million heart failure patients requiring heart transplantation surgery annually in China, costing the economy 30.7 billion dollars per year (4).

Cardiac surgeries, especially heart transplantations, rank among the most challenging surgical interventions to perform and necessitate the routine admission of patients to the ICU (5). Once in the ICU, the patients’ characteristics can have a considerable impact on ICU length of stay (ICU-LOS). Several studies and meta-analyses have revealed that renal failure can lead to a significantly prolonged LOS and higher rates of in-ICU mortality (6–8). Prolonged stays in the ICU contribute significantly to overall ICU costs. Meanwhile, the ICU-LOS varies greatly among patients. Prediction of the ICU-LOS based on patients’ clinical characteristics is beneficial for guiding the decisions of clinicians and enables hospital beds to be used more effectively. Therefore, accurate prediction of ICU-LOS is of great significance for both heart transplantation patients and the hospital.

Previous studies on the prediction of ICU-LOS have been scarce; in the majority of these, traditional static methods—such as the logistic regression model—were used (9, 10). However, these methods may be limited in terms of the number of clinical features and their linear characteristics. Nowadays, machine learning (ML) is widely used to solve biostatistical problems in medicine (11, 12). Machine learning algorithms can effectively process a large amount of data in the field of medicine. In addition, non-parametric approaches may have potential for revealing relationships otherwise obscured by non-linearities in data (13). Therefore, four machine learning models were constructed to predict ICU-LOS in heart transplantation patients based on features we summarized in our study, with the aim of providing clinical decision-making support for doctors.

## Materials and Methods

### Study Population and Data Source

Between 2017 and 2020, the data of 365 consecutive patients undergoing heart transplantation surgery for moderate (NYHA grade 3) or severe (NYHA grade 4) heart failure were collected in Wuhan Union Hospital. First, we stratified the patients into the following three groups based on ICU-LOS: the lowest quartile (25th quartile), the median (25th–75th quartiles), and the highest quartile (75th quartile). We then defined the 75th quartile of ICU-LOS (9.08 days) as the demarcation line between short and prolonged periods. The baseline characteristics are summarized in Table 1. Missing values were imputed with median of each corresponding variable. Ethics committee approval was obtained with the study adhering to the principles of the Declaration of Helsinki, and the requirement for informed consent was waived (ChiCTR2200055529).

**TABLE 1:** Baseline information.

	Level	Overall	Short	Prolong	*P*-value
*n*		365	274	91	
Recipient age (years) [mean (SD)]		47.20 (12.28)	47.08 (11.97)	47.58 (13.22)	0.734
Donor age (years) [mean (SD)]		35.58 (11.76)	34.80 (11.49)	37.95 (12.32)	**0.027**
Donor gender (%)	Male	330 (90.4)	247 (90.1)	83 (91.2)	0.926
	Female	35 (9.6)	27 (9.9)	8 (8.8)	
BMI ratio (Recipient BMI/Donor BMI) [mean (SD)]		0.99 (0.20)	1.00 (0.20)	0.99 (0.21)	0.734
Recipient blood type (%)	A	128 (35.1)	88 (36.7)	40 (32)	0.802
	B	102 (27.9)	65 (27.1)	37 (29.6)	
	AB	118 (32.3)	76 (31.7)	42 (33.6)	
	O	17 (4.7)	11 (4.6)	6 (4.8)	
Blood type match (%)	No	60 (16.4)	44 (16.1)	16 (17.6)	0.860
	Yes	305 (83.6)	230 (83.9)	75 (82.4)	
NYHA (%)	3	18 (4.9)	12 (4.4)	6 (6.6)	0.779
	4	328 (89.9)	247 (90.1)	81 (89.0)	
Cardiovascular surgery (%)	No	272 (74.5)	202 (73.7)	70 (76.9)	0.640
	Yes	93 (25.5)	72 (26.3)	21 (23.1)	
Smoking (%)	No	178 (48.8)	127 (46.4)	51 (56.0)	0.138
	Yes	187 (51.2)	147 (53.6)	40 (44.0)	
Alcohol (%)	No	252 (69.0)	187 (68.2)	65 (71.4)	0.662
	Yes	113 (31.0)	87 (31.8)	26 (28.6)	
K^+^ (mmol/L)	Normal (3.5–5.5)	231 (63.3)	170 (62.0)	61 (67.0)	0.299
	Low (<3.5)	130 (35.6)	102 (37.2)	28 (30.8)	
	High (≥5.5)	4 (1.1)	2 (0.7)	2 (2.2)	
ALT (U/L)	Normal (0–40)	254 (69.6)	188 (68.6)	66 (72.5)	0.567
	High (≥40)	111 (30.4)	86 (31.4)	25 (27.5)	
AST (U/L)	Normal (0–40)	280 (76.7)	213 (77.7)	67 (73.6)	0.509
	High (≥40)	85 (23.3)	61 (22.3)	24 (26.4)	
Cr (μ moI/L)	Normal (<106)	259 (71.0)	206 (75.2)	53 (58.2)	**0.003**
	High (≥106)	106 (29.0)	68 (24.8)	38 (41.8)	
INR (%)	Normal (0.8–1.3)	304 (83.3)	233 (85.0)	71 (78.0)	0.279
	Low (≤0.8)	4 (1.1)	3 (1.1)	1 (1.1)	
	High (≥1.3)	57 (15.6)	38 (13.9)	19 (20.9)	
BNP (pg/ml)	Normal (<100)	56 (15.3)	45 (16.4)	11 (12.1)	0.409
	High (≥100)	309 (84.7)	229 (83.6)	80 (87.9)	
LDL (mg/dl)	Normal (<3.4)	337 (92.3)	251 (91.6)	86 (94.5)	0.501
	High (≥3.4)	28 (7.7)	23 (8.4)	5 (5.5)	
Renal complication (%)	No	318 (87.1)	249 (90.9)	69 (75.8)	**<0.001**
	Yes	47 (12.9)	25 (9.1)	22 (24.2)	
Hepatic complication (%)	No	340 (93.2)	258 (94.2)	82 (90.1)	0.278
	Yes	25 (6.8)	16 (5.8)	9 (9.9)	
Hypertension (%)	No	302 (82.7)	225 (82.1)	77 (84.6)	0.699
	Yes	63 (17.3)	49 (17.9)	14 (15.4)	
Diabetes (%)	No	305 (83.6)	231 (84.3)	74 (81.3)	0.615
	Yes	60 (16.4)	43 (15.7)	17 (18.7)	
Extracorporeal circulation time (minutes) [mean (SD)]		118.53 (63.16)	113.13 (65.19)	134.78 (53.74)	**0.004**
Aortic cross clamp time (minutes) [mean (SD)]		32.75 (15.71)	31.69 (10.23)	35.93 (25.82)	**0.025**
Surgery time (minutes) [mean (SD)]		260.06 (75.48)	248.48 (64.34)	294.93 (94.05)	**<0.001**
Chest re-exploration (%)	No	354 (97.0)	270 (98.5)	84 (92.3)	**0.008**
	Yes	11 (3.0)	4 (1.5)	7 (7.7)	
IABP (%)	No	203 (55.6)	175 (63.9)	28 (30.8)	**<0.001**
	Yes	162 (44.4)	99 (36.1)	63 (69.2)	
ECMO (%)	No	346 (94.8)	271 (98.9)	75 (82.4)	**<0.001**
	Yes	19 (5.2)	3 (1.1)	16 (17.6)	
CRRT (%)	No	319 (87.4)	256 (93.4)	63 (69.2)	**<0.001**
	Yes	46 (12.6)	18 (6.6)	28 (30.8)	

*Aortic Cross Clamp Time refers to the time of aortic occlusion during the cardiac surgery.*

*Renal/hepatic complications refer to post-operative renal or hepatic damage that maybe related to the transplant as demonstrated by significant elevation of serum levels of ALT/AST or Cr. ECMO was used as arterio-venous ECMO providing both cardiac and respiratory support or as V-V ECMO, which only provides oxygenation.*

*Intra-aortic balloon pump is an important physiologic adjunct in the temporary support for the failing myocardium.*

*Continuous renal replacement therapy (CRRT) is common practice in critical care patients with acute renal failure.*

*Chest Re-exploration means that post-operative patients had emergencies like a massive hemorrhage in the thoracic cavity, compressing the pulmonary tissue and resulting in atelectasis.*

*High Cr in our study as value of creatinine above the normal range (108 μmoI/L).*

*High ALT/AST in our study as value of ALT/AST above 40 U/L.*

*The normal INR/BNP/LDL range was 0.8–1.3, <100 pg/ml and <3.4 mg/dl. Variables with p-values less than 0.05 are bolded.*

### Variable Definition and Collection

A total of 84 patient characteristics were collected for subsequent studies. These clinical features have been reported or considered by clinicians to be closely related to cardiovascular diseases. For example, there was a direct link between the level of potassium concentration and the occurrence of arrhythmia (14). Additionally, the elevated levels of ALT and AST appear in liver function abnormalities, which correlated with the prognosis (15). The collected information included the following: recipient gender; recipient age; recipient BMI; recipient blood type; donor age; donor gender; donor BMI; donor blood type; cold ischemic time; classification of NYHA heart function (NYHA); history of cardiac surgery; smoking history; alcohol history; no assistant device; implantable cardioverter-defibrillator (ICD); combined cardioverter-defibrillator (CRT-D); intra-aortic balloon pump (IABP); extracorporeal membrane oxygenation (ECMO); dopamine; angiotensin-converting enzyme inhibitors (ACEI); angiotensin receptor blocker (ARB); β-blockers (BB); red blood cell; platelet; white blood cell; neutrophil-to-lymphocyte ratio; pre-operative hemoglobin; uric acid; cholesterol; potassium ions; alanine aminotransferase; aspartate aminotransferase; albumin; total bilirubin; serum creatinine; urea nitrogen; activated partial thrombin time; international normalized ratio (INR); NT-proBNP; troponin I; free thyroid hormone; free triiodothyronine; thyrotropin; low-density lipoprotein (LDL); triglycerides (TG); respiratory complications; neurological complications; renal complications; liver damage; septic shock; hospital death; post-operative hospital stay; survival; survival time; survival for 1 year; survival for 3 months; survival for 1 month; survival for 1 year; hypertension; diabetes; hyperlipidemia; hyperthyroidism; stroke; hematocrit; extracorporeal circulation time; aortic cross clamp time; the operation time; whether to re-exploration the chest; ICU length of stay. In our study, we define matching blood type as the blood types of the recipient and the donor being exactly the same. Otherwise, the blood type is different, but it can be heterotype compatible, for convenience, we describe the former as “yes” and the latter as “no.” All clinical data were obtained from a review of the patients’ medical records. Whether a recipient was considered to have complications or a history of ECMO/chest re-exploration, etc. are based on whether this was mentioned in the progress notes or the discharge summary/diagnosis.

### Feature Extraction

We performed feature preselection using the Least Absolute Shrinkage and Selection Operator (LASSO) algorithm on the candidate features, followed by PLS-DA (Partial Least Squares-Based Discriminant Analysis) classification using the LASSO-selected features to single out the optimal features (16). The dataset was randomly split into the training set and the testing set at a ratio of 7:3.

To our knowledge, cross validation includes k-fold cross validation and leave-one-out cross validation. According to the literature, it is proposed that the leave-one-out test might overfit in small samples, whereas the K-fold cross-validation should do better (17). Meantime, the efficiency of k-fold cross validation is higher than the leave-one-out test in theory, and k-fold cross validation can be repeated k times, that means the evaluation value we get is the average of the k times results. In other words, once calculation error occurred, the results of the others will compensate, and finally output represent the level of the overall result. Then, to estimate the sample performance, we made use of fivefold cross validation. We used fivefold cross validation instead of 10-fold cross validation because at small training sample sizes (e.g., 20, 25, 30) the condition “at least one different sample per class in every fold” didn’t hold for 10-fold cross validation. The classification model was then built using the training set, with the testing set being used for prediction and performance evaluation. In the LASSO model, the minimum criterion (λ) based on five cross-validations was chosen.

In addition, in our study, we use up-sampling to process the data, which can randomly samples so that replacement from the smallest class is the same size as the largest class (18). And we also avoid complex models with many parameters, thus limiting their generalization and possibility of overfitting. Furthermore, we use linear model and tree-based model to select the features, to build a better model with the least features to ensure not to be fooled by overfitting (19).

### Linear Regression Model

Linear Regression (LR) is a standard statistical generalized linear model method used in data mining, automatic disease diagnosis, economic forecasting, and other broad applications. The algorithm is essentially a conventional two-category model, with the object’s category determined by inputting the object’s attribute sequence. To classify the data, the model assumes that the data follows the Bernoulli distribution and employs the method of maximizing the likelihood function to solve the parameters with gradient descent. In our study, a multivariable LR model was built using the function glm of the R package stats. Odds ratios were calculated for each risk factor by exponentiating the LR coefficients. Odds ratios less than 1.0 indicate a decreased risk, while odds ratios greater than 1.0 indicate an increased risk, and the *p*-value of <0.05 was considered significant.

### Machine Learning Models

Four classic machine learning models with fivefold cross-validation were developed to predict ICU-LOS; namely, Naive Bayes (NB), Random Forest (RF), Support Vector Machines (SVM), and XGboost. All machine learning models were constructed using Random Forest, XGboost, and caret packages in the R programming language (version 3.3.1). As an extra level of precaution, models were trained and tested by two different team members (LY and KW) to ensure that models were never accidentally trained using held-out test data.

### Random Forest

The random forest method is a machine learning technique that mixes many decision trees to create a single classification model. The random forest approach generates a forest of multiple decision trees by selecting various dividing characteristics and training samples. When predicting unknown samples, each tree in the forest is trained to make decisions, significantly increasing prediction accuracy compared to a single decision tree. After statistically assessing the decision outcomes, the classification with the most votes are recognized as the official classification result.

### Naive Bayes

Naive Bayes classifier is a highly scalable supervised learning technique. Bayesian reasoning is based on probability to derive conclusion about the ideal decision’s probability distribution. It has been effectively implemented in various scientific areas and consistently performs well even when just a few variables are considered.

### Support Vector Machines

Support vector machines is a machine learning approach that is based on statistical learning theory. A support vector machine aims to minimize generalization error by creating a hyperplane in a high-dimensional space and utilizing a maximum margin to separate feature vectors belonging to distinct classes. When a support vector machine is used for linear classification, an n–1 dimensional hyperplane is used, where n is the dimension of the data.

### eXtreme Gradient Boosting

eXtreme Gradient Boosting is one of the most extensively used machine learning classifiers in bioinformatics. It is based on a tree model that classifies using a boosting method, according to the literature, it can also perform a good fit when randomly reducing the sample size to 100 (20). Regularization elements are added to the cost function to minimize the model’s complexity and to prevent overfitting. Additionally, the parallel computing function is enabled by the XGboost algorithm, which significantly accelerates calculation.

### Shapley Additive Explanations

Shapley additive explanations is a recently developed technique that aims to interpret black box machine learning models. Lundberg et al. examined several contemporary algorithms for determine feature importance and showed that they belonged to the same class of measures, then unified them into the SHAP framework (21). Most previous machine learning algorithms provide predictors with global feature importance, and it is difficult to interpret each prediction case. However, the SHAP technique calculates the contribution of each input variable in each decision of a machine learning model. We obtained SHAP values by executing package “shap” to interpret model predictions (22).

### Statistical Analyses

The machine learning models included random forest (RF), naive Bayes (NB), support-vector machines (SVM), and XGBoost. Linear regression (LR) and machine learning models were performed using R software (4.1.1) and Python (Version 2.8). Then, the performance of these machine learning methods was measured by calculating the AUC—the area under the ROC (relative operating characteristic) curve.

## Results

### Patients’ Characteristics

**FIGURE 1:**
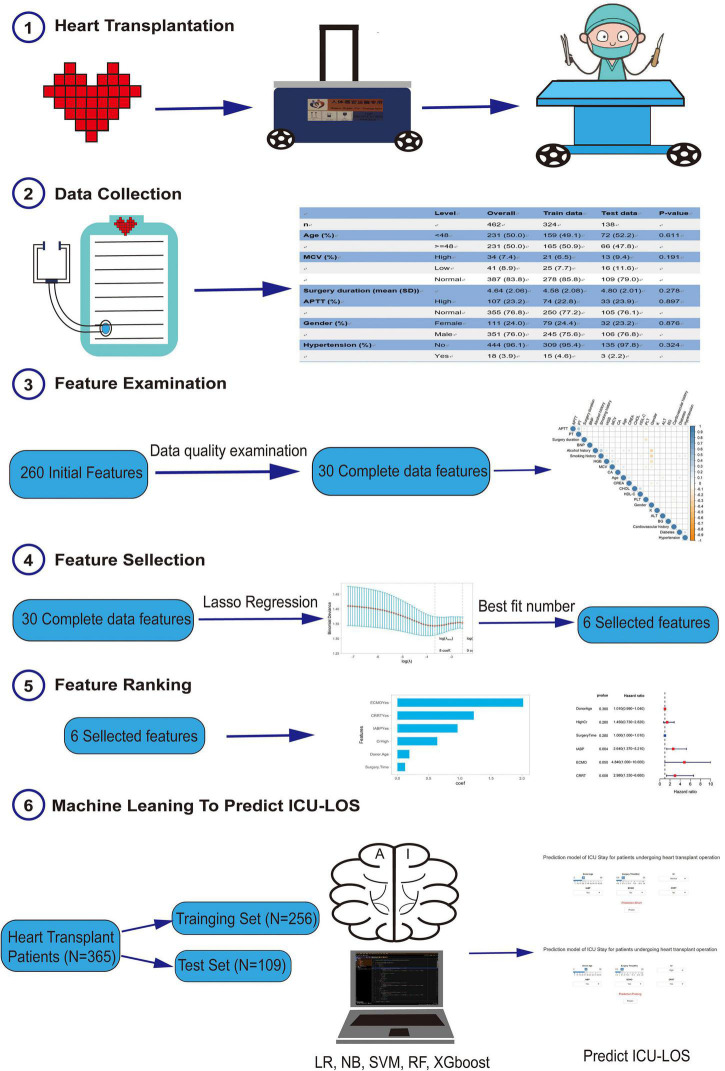
The study flow diagram.

As shown in Figure 1, 365 patients were enrolled in this study. The baseline characteristics of these patients are shown in Table 1. The median age of the recipient patients was 47.2 years, while the median age of the donors was 35.58 years. 330 (90.4%) of the donor patients were men, and the average surgery duration was 260.06 min. Among this cohort, 47 (12.9%) had renal complications, 25 (6.8%) had hepatic complications, 11 (3%) had undergone chest re-exploration, and 19 (5.2%) had undergone extracorporeal membrane oxygenation (ECMO).

### Feature Selection

**FIGURE 2:**
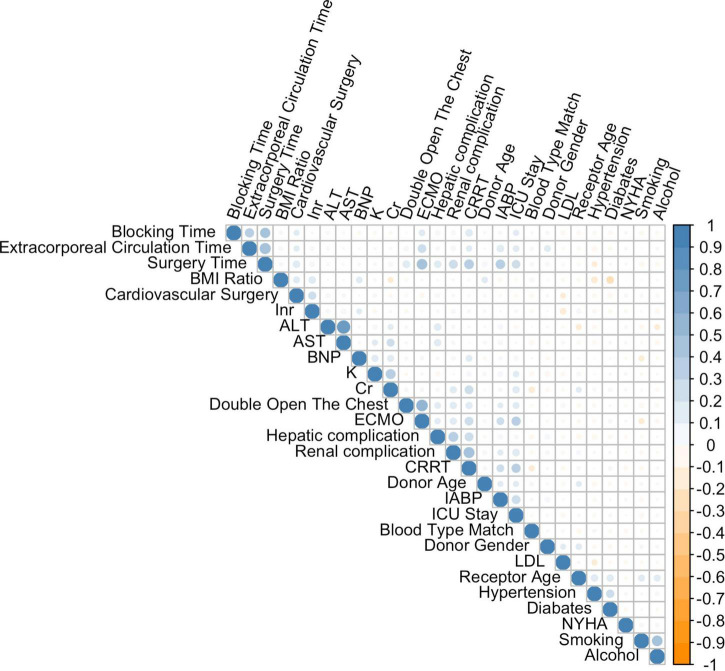
Correlations among variables. The color scale ranges from blue (coefficient of 0) through white (coefficient of 0.5) to orange (coefficient of 1).

**FIGURE 3:**
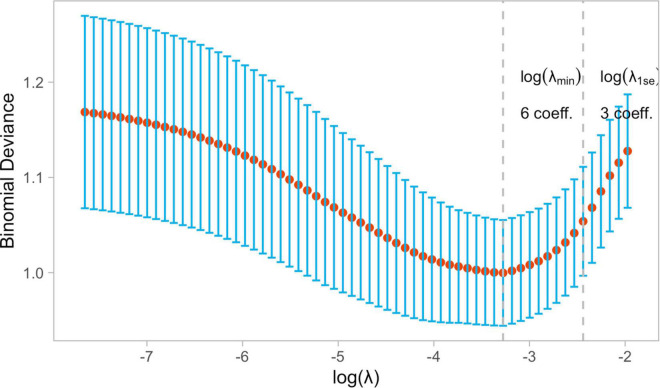
The Least Absolute Shrinkage and Selection Operator (LASSO) binary logistic regression model was used to select clinical features. The binomial deviance metrics (the y-axis) were plotted against log(λ) (the bottom x-axis) and the LASSO model used fivefold cross-validation *via* minimum criteria.

**FIGURE 4:**
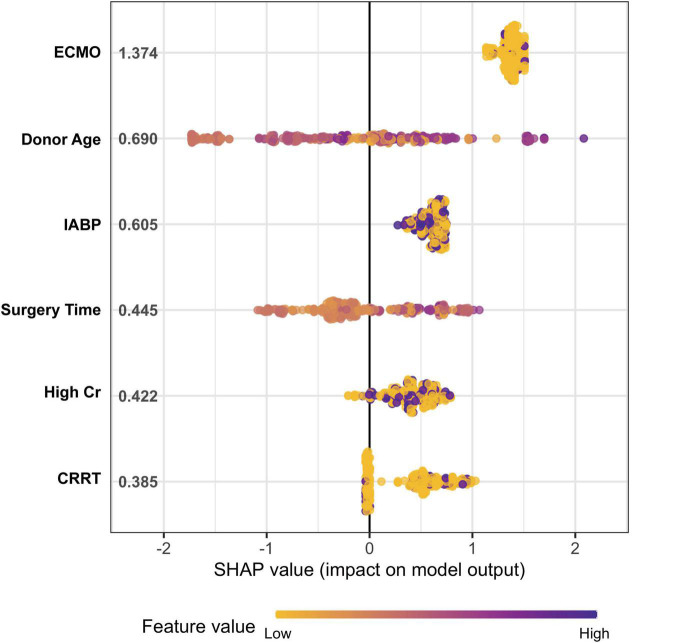
The importance rank and hazard ratio of selected features using SHAP. The importance rank of selected features. Each point in the figure is a feature value of a particular training example. The color of the point represent the feature value and the X-axis position of the point is its SHAP value. The features are ranked by the sum of SHAP value magnitudes over all samples.

These patients were randomly placed into a training set (*N* = 256) and a test set (*N* = 109), which were used to train, optimize and evaluate models (Table 2). The results revealed that variables *p*-values for the training and validation sets were greater than 0.05, which indicated that there has no significant differences between training and test dataset variables. Figure 1 presents a flow diagram depicting the sampling strategy for this study. 30 clinical features were selected as crucial variables by machine learning methods. We performed a correlation analysis between these features (Figure 2). Besides ALT and AST, no additional significant correlations were identified through correlation analysis. We then used LASSO regression (LR) to identify six variables that were relevant for algorithm development (Figure 3). Meantime, we include all features to measure the error rate under fivefold cross-validation, as the features continue to be deleted, the machine learning model based on just six features has the lowest error rate (Supplementary File 1, and we showed in Supplementary File 2 that an open-source code can run on R 4.1.1 software). Interpreting model predictions becomes more and more important in the field of machine learning, especially for deep learning. An outstanding approach has been proposed, which used SHAP values as a unified measure of feature importance (23). Each point in the figure is a feature value of a particular training example. The color of the point represent the feature value and the X-axis position of the point is its SHAP value. The features are ranked by the sum of SHAP value magnitudes over all samples. Figure 4 shows the detailed impact of each feature to the model output in dataset using SHAP. Compared with XGBoost feature importance result, the rank of feature importance is different between two methods, but the use of ECMO continues contributed the greatest weight to the result of ICU-LOS (Supplementary File 3). Meantime, SHAP analysis of feature importance for prediction of ICU-LOS (the severity of patients’ conditions) was consistent with current medical knowledge. The use of IABP, CRRT can both to be bad prognostic features of a patient undergoing heart surgery (24). Increases in Cr (renal complications) can be caused by prolonged surgery time, so it makes sense that this would be an important predictive feature. In order of importance, these variables were: (1) the use of extracorporeal membrane oxygenation (ECMO); (2) donor age; (3) the use of an intra-aortic balloon pump (IABP); (4) length of surgery; (5) high creatinine (Cr); and (6) the use of continuous renal replacement therapy (CRRT) (Figure 4).

**TABLE 2:** The entire dataset was split into a training set and testing set (7:3).

	Level	Overall	Train data	Test data	*P*-value
*n*		365	256	109	
Donor age [mean (SD)]		35.58 (11.76)	35.86 (11.94)	34.94 (11.35)	0.493
Cr (%)	High	106 (29.0)	71 (27.7)	35 (32.1)	0.473
	Normal	259 (71.0)	185 (72.3)	74 (67.9)	
Surgery time [mean (SD)]		260.06 (75.48)	259.39 (71.72)	261.64 (83.98)	0.795
IABP (%)	No	203 (55.6)	138 (53.9)	65 (59.6)	0.372
	Yes	162 (44.4)	118 (46.1)	44 (40.4)	
ECMO (%)	No	346 (94.8)	246 (96.1)	100 (91.7)	0.146
	Yes	19 (5.2)	10 (3.9)	9 (8.3)	
CRRT (%)	No	319 (87.4)	219 (85.5)	100 (91.7)	0.144
	Yes	46 (12.6)	37 (14.5)	9 (8.3)	
ICU stay (%)	Prolong	91 (24.9)	64 (25.0)	27 (24.8)	1.000
	Short	274 (75.1)	192 (75.0)	82 (75.2)	

### Performance of Intensive Care Unit Length of Stay Prediction Models

**FIGURE 5:**
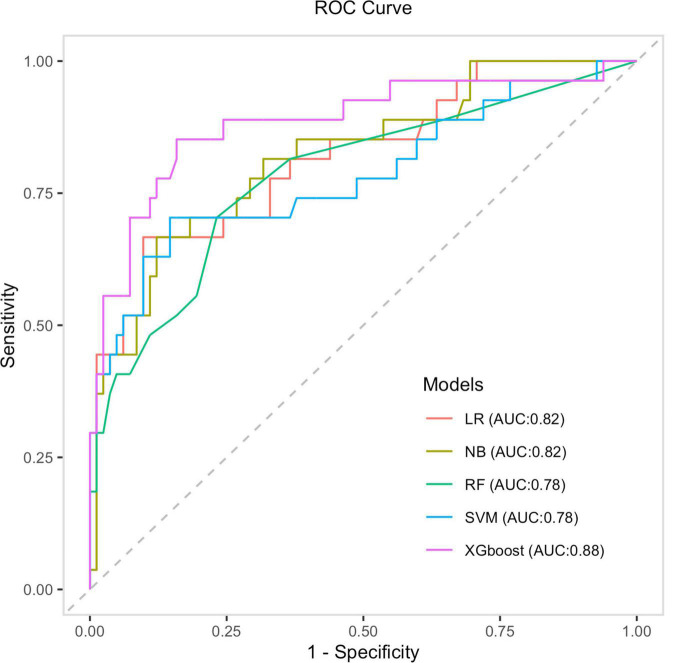
The ROC curves of four machine learning models and the LR model.

**FIGURE 6:**
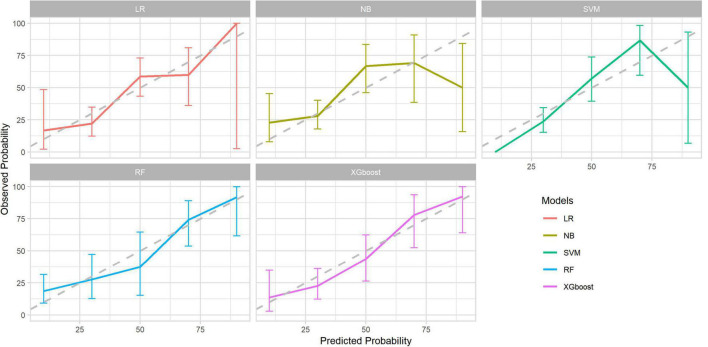
Calibration curve of the four machine learning models and LR model.

After identifying the six variables, the receiver operating characteristics (ROC) for predicting the length of ICU-LOS in heart transplantation patients by using machine learning models were determined; these are presented in Figure 5. As shown in Table 3, the AUC of the XGBoost model is 0.88. The XGBoost model (accuracy: 0.87; sensitivity: 0.98; specificity: 0.51; positive predictive value: 0.86; negative predictive value: 0.93) significantly outperformed the conventional LR (AUC = 0.84) and three other machine learning models (NB: AUC = 0.82; RF: AUC = 0.80; SVM: AUC = 0.81). The sensitivity of each model also confirmed the robustness of our model results. The predicted probability of the LR, NB, SVM, RF, and XGBoost models is shown in Figure 6.

**TABLE 3:** Performance of machine learning models.

Model	Accuracy	Sensitivity	Specificity	PPV	NPV	AUC-ROC	AUC-Lower	AUC-Upper
LR	0.8440	0.9878	0.4074	0.8351	0.9167	0.8159	0.7784	0.8635
RF	0.8073	0.9634	0.3333	0.8144	0.7500	0.7827	0.7253	0.8202
NB	0.8257	0.9634	0.4074	0.8316	0.7857	0.8205	0.7753	0.8756
SVM	0.8165	0.9878	0.2963	0.8100	0.8889	0.7839	0.6981	0.8397
XGboost	0.8780	0.9883	0.5190	0.8699	0.9318	0.8828	0.8572	0.9284

### Online Prediction Tool

**FIGURE 7:**
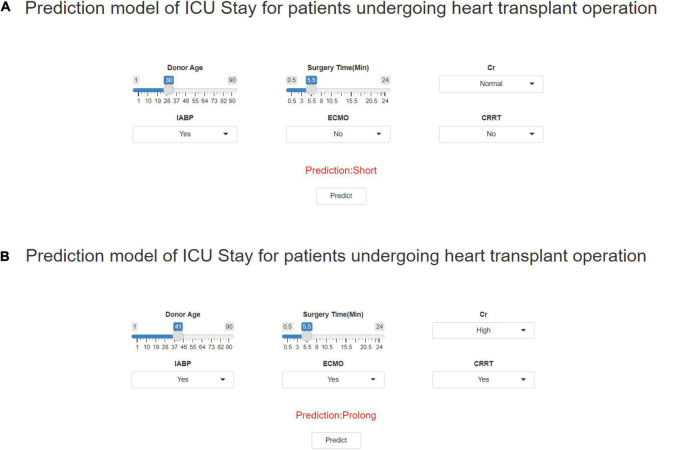
Outcomes were generated by the prediction tool for two fictitious case scenarios. Enter the values of six key variables to predict the risk of ICU-LOS. **(A)** Has a short ICU-LOS and **(B)** Has a prolonged ICU-LOS.

The final model was incorporated into an online open-access platform prediction tool, allowing users to calculate the ICU-LOS of patients following heart transplantation surgery^1^ based on six selected features. Figure 7 displays the results generated by the prediction tool for two case scenarios. We analyzed the training time to build an explainable XGboost model using the whole training dataset. The training process was completed within 50 min to obtain the final multiclass model. During the validation process, the execution time required for one case was 30 ms using our platform without a graphic processing unit.

## Discussion

With the continuous development of societies, people’s living standards and quality of life have been significantly improved; however, most countries—especially developed countries—are prone to population ageing (25), with heart failure being recognized as a major aging-associated disease (26, 27). However, with the exception of heart transplantation surgery for end-stage heart failure, no curative treatment has yet become available (28). After the heart transplantation procedure, post-operative patients are admitted to the ICU as a matter of course. Prolonged ICU-LOS brings further financial risk, as well as risks associated with morbidity and mortality (29). Premature ICU discharge may potentially expose patients to the risks of unsuitable treatment, which further leads to preventable mortality (30). However, it remains a difficult task for clinicians to accurately predict the ICU-LOS due to the complexity of characteristics presented by different patients (31).

As electronic health records have become more prevalent, we have been able to harness the power of machine learning to accurately predict the length of stay. Machine learning models have been developed to predict the length of stay in the ICU after heart surgery (32). Other studies have also used these models to predict mortality and disease severity in the ICU (33). A previous study has trained a support vector machine (SVM) model to forecast patient survival and length of stay using data from 14,480 patients (34). The AUC of prolonged ICU length of stay was 0.82. Mani et al. reported that a hidden Markov model also predicted ICU length of stay with reasonable accuracy (35). Although there have been several models to predict ICU-LOS, most of them have focused on patients undergoing cardiac surgery and thus may not be suitable for heart transplantation surgery patients. Our study, to the best of our knowledge, is the first to predict ICU-LOS in heart transplantation patients using four machine learning methods in one single ICU. Also, by using this prediction algorithm for ICU-LOS based on patients’ clinical features, we can direct the clinicians’ attention toward patients who are most at risk.

Intensive care unit length of stay (LOS) is a well-established measure of ICU resource use and performance. In only 53% of cases can the ICU-LOS of patients be adequately predicted by ICU physicians (36), and the reason for a longer ICU-LOS remains unclear. Our primary purpose in this article was to precisely identify patients’ ICU-LOS in order to best utilize medical resources and give practical advice for clinicians. In this study, we applied 84 basic clinical characteristics to LASSO algorithms and determined the six most important clinical features in increasing the ICU length of stay. The six clinical features are (1) the use of extracorporeal membrane oxygenation (ECMO); (2) donor age; (3) the use of an intra-aortic balloon pump (IABP); (4) length of surgery; (5) high creatinine (Cr); and (6) the use of continuous renal replacement therapy (CRRT). Indeed, it is not surprising that ECMO, IABP, and CRRT are critical in increasing the LOS in the ICU. This also clearly demonstrates that quality criteria for feature selection is accurate and stable. Meanwhile, in addition to these recognized risk factors, other factors and models still have some potential influence. For example, the value of Cr indicates the degree of renal dysfunction, which directly affects the speed of post-operative recovery and may bring about an increase in operating time and, consequently, medical expenses. Prolonged surgery time was found to cause an increase in the aortic cross clamp time and the time to return to high PaO2, causing damage to several organs and increasing the length of stay in the ICU. In addition, the quality of the donor organ depends on donor-related factors, such as donor age, which ultimately affects the prognosis of the transplantation (37, 38). Also, in 2019, a nationwide report from South Korea revealed that donor age has a close relationship to conditional mortality in heart transplantation patients (39). Furthermore, abnormal blood creatinine levels suggest internal environment disorder and impairment of renal function, indicating a worse state of patients (40–42). These results showed that changes in pre-operative conditions may be predictive of clinical endpoints (43).

Based on these results, prediction models were developed using four machine learning methods. It was observed that, among these four methods, the XGBoost classifier exhibited stronger diagnostic power for the predictions, with an area under the ROC curve of 0.88. Also, its effect was significantly better than of the traditional linear regression methods (LR: ROC = 0.81). This implies that the use of machine learning—especially XGBoost—in making predictions based on the pre-operative patients’ characteristics will enable us to determine patients’ ICU-LOS earlier, allocate medical resources accordingly, and optimize treatment procedures. For the convenience of clinicians, a predictive model for calculating ICU-LOS in heart transplantation patients was established; it was displayed using a webpage calculator for convenient clinical application (see text footnote 1).

However, this study has some limitations. A major limitation is that the research data came from one single ICU with a small number of patients, and there may be some bias caused by regional factors. In medical research, relatively small data sets are common, which often leads to concerns about the stability of models (19). Because of this, we made sure to use simple models and select the features accordingly so that the results would not suffer from the problem of overfitting. This issue can also be solved by external validation; however, due to the particularity of clinical data, clinical details of heart transplantation patients in other centers were not easily accessible. An additional limitation is that the published articles only gave us access to the researchers’ inferred data and not the raw data; thus, it was impractical to perform an external validation in this study. A further limitation is that we could not provide a causal relationship between factors such as increased surgery duration or impaired renal function and the observed prolonged ICU treatment. However, a goal-directed study should be able to provide such a relationship.

In summary, the overall results revealed the XGBoost model to be the superior model of classification for predicting heart transplantation patients’ ICU-LOS (AUC = 0.88) in comparison to logistics regression (LR) and three other machine learning models (NB, RF, and SVM). Also, six clinical features were identified. It is reasonable to assume that the coming era of big data and personalized medicine will see a significant increase in machine learning applications for assisting clinical decisions.

## Data Availability Statement

The original contributions presented in the study are included in the article/Supplementary Material, further inquiries can be directed to the corresponding authors.

## Ethics Statement

Ethics committee approval was obtained with the study adhering to the principles of the Declaration of Helsinki, and the requirement for informed consent was waived (ChiCTR2200055529).

## Author Contributions

KW conceived and designed the study and wrote the manuscript. KW, LY, and WL collected and analyzed the data. CJ, NW, and QZ revised the manuscript. JS and ND reviewed and edited the manuscript. All authors participated in discussions and approved the final manuscript.

## Conflict of Interest

The authors declare that the research was conducted in the absence of any commercial or financial relationships that could be construed as a potential conflict of interest.

## Publisher’s Note

All claims expressed in this article are solely those of the authors and do not necessarily represent those of their affiliated organizations, or those of the publisher, the editors and the reviewers. Any product that may be evaluated in this article, or claim that may be made by its manufacturer, is not guaranteed or endorsed by the publisher.

## Footnotes

1
https://wuhanunion.shinyapps.io/PredictICUStay/

## Supplementary Material

Supplementary File 1 Fivefold cross-validation for selecting appropriate features.

Supplementary File 2 An open-source code for estimate error rate in decreasing features.

Supplementary File 3 The importance rank and hazard ratio of selected features using XGBoost algorithms. **(A)** The importance rank of selected features; **(B)** The hazard ratio of selected features.

## Abbreviations

ICU-LOS: ICU length of stay
LASSO: the least absolute shrinkage and selection operator
AUC-ROC: the area under the receiver operating characteristic
Cr: creatinine
ECMO: extracorporeal membrane oxygenation
CRRT: continuous renal replacement therapy
IABP: intra-aortic balloon pump
PPV: positive predictive value
NPV: negative predictive value
RF: Random Forest
NB: Naive Bayes
SVM: Support Vector Machines
LR: linear regression
XGboost: The eXtreme Gradient Boosting algorithms
SHAP: Shapley Additive explanations.
