# Supplementary material for: Comparison of Four Machine Learning Techniques for Prediction of Intensive Care Unit Length of Stay in Heart Transplantation Patients
Source: Front Cardiovasc Med. 2022 Jun 21;9:863642. doi: 10.3389/fcvm.2022.863642 (PMC9253610; doi:10.3389/fcvm.2022.863642)
Supplement: Supplementary File 1 — Fivefold cross-validation for selecting appropriate features. [file Presentation_1.zip › Image_2.DOCX]

1. *# Data Precessing*
2. library(ggplot2)
3. library(magrittr)
4. *# Get data from file*
5. source('modify data.R',encoding = 'utf-8')
6. data <- get_data() %>% data.frame()
7. out_col = 'ICU.Stay'
8. *# Feature Selecting -- RFCV ------------------------------------*
9. library(randomForest)
10. set.seed(13433)
11. result <- replicate(5, rfcv(data %>% .[,setdiff(names(.),out_col)], data[[out_col]],cv.fold = 5, step=1.1), simplify=FALSE)
12. result %<>% sapply('[[', 'error.cv') %>% data.frame(check.names = F) %>% setNames(c("err1", "err2", "err3", "err4", "err5"))
13. result %<>% mutate(errmean = rowMeans(.)) %>% mutate(num = as.numeric(rownames(.)))
14. optim_num = result%>%.[which(.$errmean==min(.$errmean)),'num']
15. ggplot(result, aes(x = num))+
16. geom_line(aes_(y = as.name(names(result[6])), colour = 'Mean error'),lwd=1.2)+
17. scale_y_continuous(limits=c(0,0.3))+
18. scale_colour_manual("", breaks = c('Mean error'),values = c('#e64602'))+
19. geom_vline(xintercept = optim_num, colour='gray', lwd=0.5, linetype="dashed") +
20. annotate("text", x = optim_num - 1, y = 0.1, label=paste0("Optimal = ",optim_num),size=3,hjust=0)+
21. labs(
22. title = "Five-fold cross-validation for selecting appropriate features",
23. x = "Number of variables",
24. y = "Error rates of cross-validations",
25. )+
26. coord_trans(x ="reverse")+
27. scale_x_continuous(breaks = seq(1,length(colnames(data)),2)) +
28. theme(text = element_text(size=10),
29. panel.background = element_rect(fill = "transparent", colour = NA),
30. panel.border = element_rect(fill = NA,colour = "grey70", size = rel(1)),
31. panel.grid = element_blank(),
32. axis.ticks = element_line(colour = "grey70", size = rel(0.5)),
33. complete = TRUE,
34. legend.position=c(0.2,0.2),
35. legend.key = element_rect(colour = "transparent",fill = NA))
