# Supplementary figures and images for: Comparison of Four Machine Learning Techniques for Prediction of Intensive Care Unit Length of Stay in Heart Transplantation Patients
Source: Front Cardiovasc Med. 2022 Jun 21;9:863642. doi: 10.3389/fcvm.2022.863642 (PMC9253610; doi:10.3389/fcvm.2022.863642)

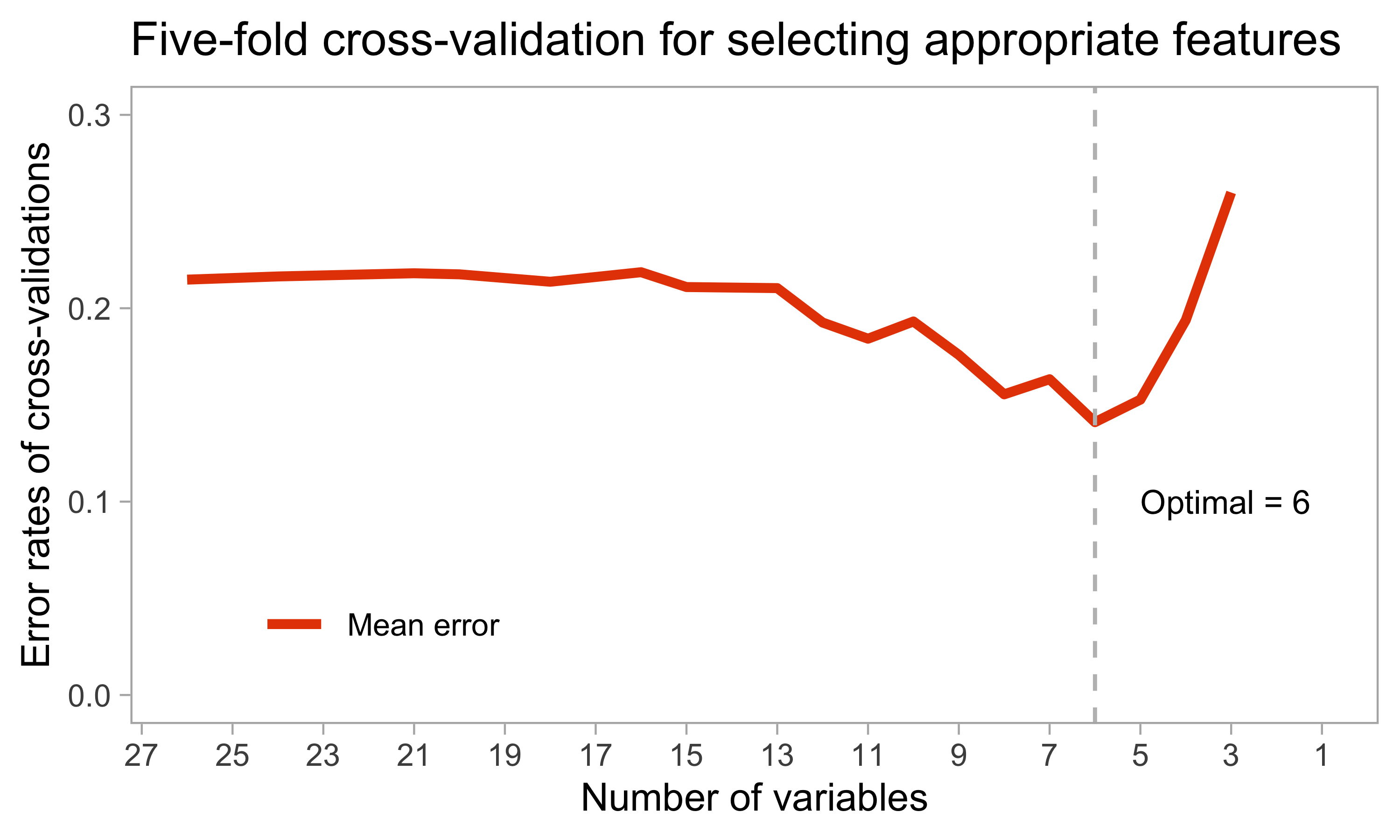

Supplement: Supplementary File 1 — Fivefold cross-validation for selecting appropriate features. [file Presentation_1.zip › Image_1.PNG]

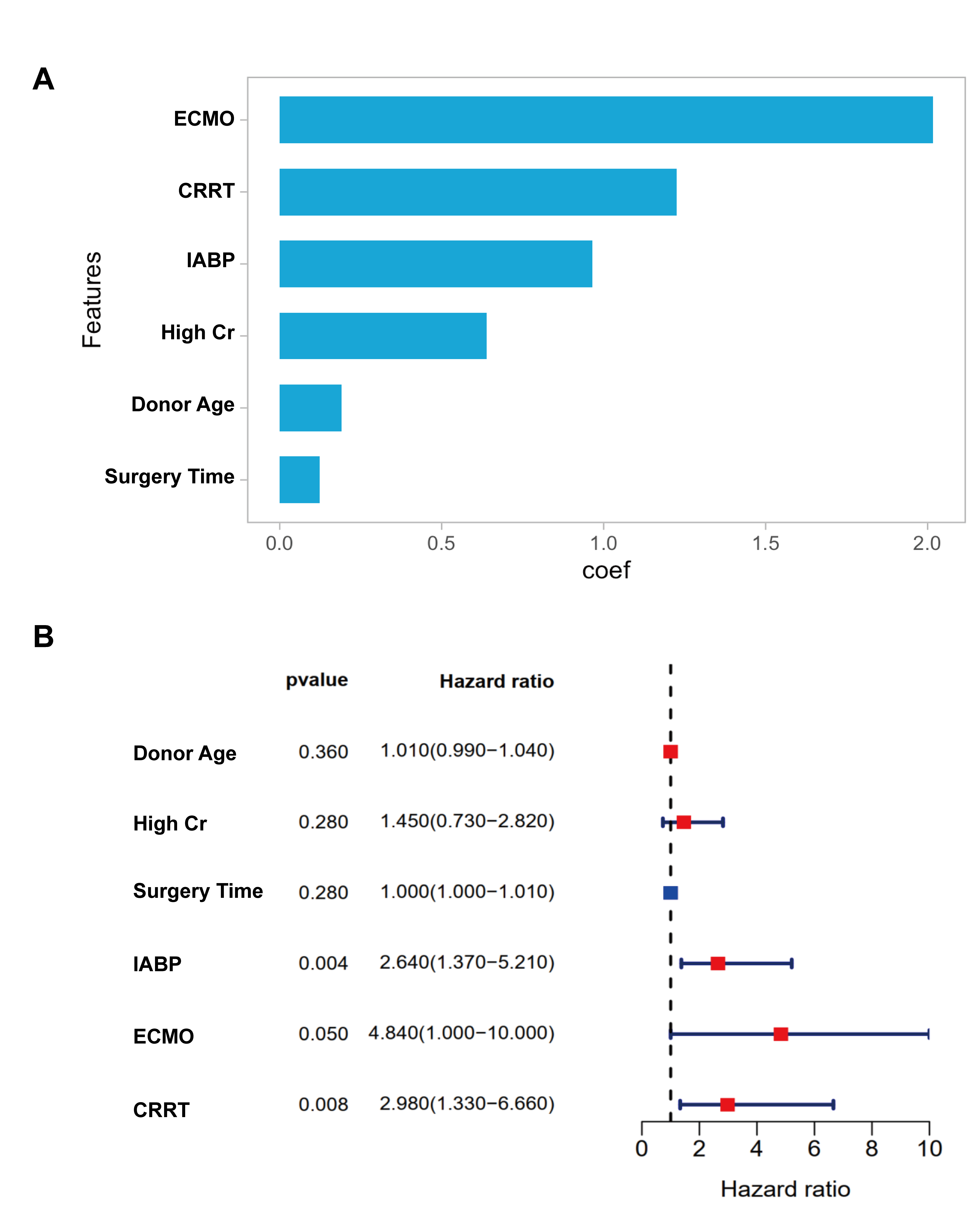

Supplement: Supplementary File 1 — Fivefold cross-validation for selecting appropriate features. [file Presentation_1.zip › Image_3.tif]
